# Whole-genome sequencing and phenotyping of neglected and underutilized vegetable melons from the Salento diversity centre (Southern Italy)

**DOI:** 10.3389/fpls.2025.1644621

**Published:** 2025-08-19

**Authors:** Marzia Guerriero, Francesco Arcieri, Chiara Delvento, Gaetano Giudice, Marco Santo Cannarella, Giancarlo Mimiola, Giuseppe Cavallo, Luigi Ricciardi, Concetta Lotti, Stefano Pavan

**Affiliations:** ^1^ Department of Soil, Plant and Food Sciences, University of Bari Aldo Moro, Via Amendola 165/A, Bari, Italy; ^2^ Centre International De Hautes Études Agronomiques Méditerranéennes (CIHEAM), Valenzano (Bari), Italy; ^3^ Department of Agricultural, Food and Environmental Sciences, University of Foggia, Foggia, Italy

**Keywords:** cucumber melon, snake melon, whole-genome resequencing, diversity, private alleles

## Abstract

The species *Cucumis melo* L. includes two neglected and underutilized vegetable crops, cucumber melon (*C. melo* subsp. *melo* var. *chate*) and snake melon (*C. melo* subsp. *melo* var. *flexuosus*). In particular, cucumber melon was highly popular in Mediterranean civilizations during Antiquity and the Middle Ages, whereas today its cultivation is mostly confined to the Salento area of southern Italy. Here, we describe the collection and characterization of thirteen cucumber melon and two snake melon populations from Salento. Whole-genome resequencing of DNA pools was performed to investigate genetic diversity within and among populations. The cucumber melon population UBGCMC111, most widely cultivated and marketed, exhibited the lowest heterozygosity, possibly reflecting more intense selection by farmers. Hierarchical clustering revealed genetic divergence of UBGCMC111 and UBGCMC053, the latter originating from a unique area of Salento with linguistic and cultural ties to Greek heritage. Despite some unique patterns of variation, snake melons clustered together with cucumber melons, suggesting overall genetic similarity. A total of 1,307 alleles were fixed and private to different populations under study, potentially valuable for their traceability. Some of them were associated with genes possibly underlying deeply grooved and pale green pepo phenotypes of the populations UBGCMC111 and UBGCMC124, respectively. Replicated field trials enabled germplasm characterization and the selection of agronomically superior populations. Overall, this study safeguards valuable *C. melo* genetic diversity from further genetic erosion. Additionally, it provides genomic and phenotypic data laying a foundation for integrating unexplored genetic resources into mainstream agrifood systems and breeding programs.

## 1 Introduction

Melon (*Cucumis melo* L.) is an extraordinarily polymorphic species, comprising 15 cultivated and wild botanical groups classified into the two subspecies *melo* and *agrestis* (Pitrat, 2008). Sweet melons, which now dominate the global market, emerged relatively recently in Europe during the Middle Ages (Paris et al., 2012). In contrast, vegetable melons, which do not accumulate sugars during ripening and are typically consumed at the immature stage, were already popular in ancient Mediterranean civilizations. However, their cultivation is now limited to small, localized areas, making them neglected and underutilized crops (Paris et al., 2012).

Cucumber melon (*Cucumis melo* var. *chate*) is a vegetable melon that produces tender, crispy, cucumber-like pepos with an approximate width-to-length ratio of 1:2. Its epicarp, ranging in color from pale to dark green, is often covered with soft hairs. Cucumber melons were already depicted in wall paintings from Ancient Egypt (Keimer, 1924; Sabato et al., 2019). In Biblical times, they were popular in Judea with the name *qishu’im* (Paris et al., 2008). During the Middle Ages, their presence in Italy is documented through herbal iconography (Paris et al., 2011). Today, cucumber melon cultivation is largely confined to the Salento area of the Apulia Region (Southern Italy), where it is known by the local names of *meloncella* and *spuredda* (Pavan et al., 2017; Lija and Beevy, 2021). In fact, the cucumber melon is so popular in Salento that cucumber (*Cucumis sativus*) is virtually absent from local dietary habits (Laghetti et al., 2008). Recently, the cucumber melon from Salento was recognized as an Italian Traditional Agri-food Product (Didonna et al., 2023). In addition, a population largely cultivated in the municipality of Leverano, within Salento, known as *meloncella striata* due to its deeply grooved pepos, has been expanding in both cultivation and market presence, reaching large-scale retail distribution. Finally, the cucumber melon has recently gained significant international attention, highlighting its growing appeal beyond its traditional region of cultivation (Roach, 2023).

Snake melon (*C. melo* var. *flexuosus*) is another type of vegetable melon, characterized by slender pepos with a width-to-length ratio ranging from 1:3 to 1:9 (Ali-Shtayeh et al., 2017). It is the most frequently mentioned cucurbit in classical literature (Paris et al., 2012), described by the Roman writers Pliny and Columella (Janick et al., 2007) and depicted in mosaics and paintings of the Roman and Byzantine empires (Omari et al., 2018). Nowadays, the snake melon is occasionally cultivated in the Mediterranean region. In Salento, it is typically grown in the municipality of San Donato, where it is known as *cucummaru*.

To date, limited information is available on genetic and phenotypic variation of vegetable melons. This would be extremely useful to select populations better suited for cultivation and breeding. Additionally, the identification of peculiar genetic features, such as private allelic variants, could be highly valuable for the traceability and protection of specific populations through quality labels.

High-throughput sequencing and genotyping technologies enable the genetic characterization of plant germplasm collections (Pavan et al., 2021; Delvento et al., 2023). However, sequencing several individuals of open-pollinated populations, necessary to obtain accurate estimates of population genetic parameters, is still, in most cases, economically unfeasible (Correa Abondano et al., 2024). Pool-seq, i.e. the sequencing of pooled DNA from several individual, is a cost-effective approach to infer genome-wide population genetic parameters, such as allele frequencies, genetic diversity and selection signatures (Kofler et al., 2011; Schlötterer et al., 2014), and to quantify the extent of genetic differentiation among populations. Pool-seq has been successfully applied to study genetically heterogeneous populations of different crops (e.g. Correa Abondano et al., 2024; Dziurdziak et al., 2021).

Here, we report the establishment of a collection of open-pollinated, farmer-maintained vegetable melon populations cultivated in Salento. Pooled-seq whole-genome resequencing was performed to study inter- and intra-population diversity. Genetic analysis was complemented by replicated field trials, together providing valuable information for the valorization of these crops.

## 2 Materials and methods

### 2.1 Plant material and field trials

Exploratory missions were conducted across the Salento area, in search of farmer-maintained, open-pollinated populations. These were individually multiplied at the experimental farm ‘P. Martucci’ of the University of Bari Aldo Moro (UNIBA) (41°01’22.1’’ N, 16°54’21.0’’ E), using structures with isolation nets to prevent the access of pollinators. After multiplication, seeds were partially dehumidified to about 6% moisture content and placed in long-term storage chambers (-20°C) at the UNIBA Genebank.

The same populations were evaluated in field trials carried out in 2023 and 2024 at the same experimental farm. Two-week-old seedlings were transplanted on June 4, 2023, and on July 6, 2024, according to a randomized block design with three replicates, with each experimental unit consisting of five plants 0.6 m apart along the row. Experimental units were spaced 1.5 m within and between rows. Crop management was carried out according to local practices, including base fertilization with diammonium phosphate and irrigation. Pest and pathogen management was performed using single applications of deltamethrin, acetamiprid, and a copper-based fungicide. Additionally, manual and mechanical weeding were conducted during the crop cycle.

### 2.2 DNA extraction and sequencing

Genetic analysis was performed on 15 vegetable melon populations. For each of them, approximately equivalent amounts of leaf tissue were sampled from 12 individuals and used for a single DNA extraction. This was done using the Plant Genomic DNA Mini kit (Geneaid), following the manufacturer’s instructions. DNA quality and concentration were then checked by electrophoretic analysis on agarose gel (0.8%) and spectrophotometry, using the Qubit 3.0 fluorometer (Life Technologies). Whole-genome resequencing was performed using the Illumina NovaSeq PE150 platform, which provides paired-end sequencing with a read length of up to 150 base pairs.

### 2.3 Variant calling, quality control and SNP filtering

The sequences obtained were aligned against the reference genome of the *C. melo* cultivar AY_1.0 (NCBI identification code GCF_025177605.1; genome size = 438.3 Mb). A variant call format (vcf) file was then obtained using FreeBayes (Garrison and Marth, 2012), deactivating the priors related to the Hardy-Weinberg equilibrium condition, activating the group mode, and applying filters for minimum base quality and minimum quality mapping above 30. Additional filters were applied with vcftools (Danecek et al., 2011), for the selection of polymorphisms with mean read depth ranging from 20.9x and 42.3x, corresponding to the mean sample read depth ± 3sd. Additional filters were minimum quality score of 30 and less than 5% missing genotype calls.

### 2.4 Assessment of genetic diversity

The R package poolfstat (Hivert et al., 2018) was used to estimate population heterozygosity from allele depth information available in the vcf file. The same package was also employed to perform principal component analysis (PCA) and quantify genetic distances among all possible population pairs based on Wright’s F_ST_ index. Finally, the pheatmap R package (Kolde, 2019) was used to generate a heatmap and a dendrogram from the F_ST_ matrix, with clustering performed using the complete linkage method.

### 2.5 Identification and annotation of alleles privately fixed in individual populations

A custom R script was used to estimate allele frequencies for each locus and population, starting from allele depth information available in the vcf file. Then, alleles privately fixed in individual populations (i.e. with a frequency of 1 in one population and 0 in all the others) were extracted. The Variant Effect Predictors (VEP) tool (McLaren et al., 2016) was used to annotate genetic variants and predict their effect, using information available for the *C. melo* cultivar AY_1.0 public assembly (NCBI identification code GCF_025177605.1).

### 2.6 Phenotypic characterization

The following seven descriptors defined by the Italian Working Group on Agricultural Biodiversity (GIBA) for the species *Cucumis melo* L. were recorded: fruit color intensity, spot density, spot site, peduncular site shape, distal end shape, groove presence/absence, and groove depth. In addition, the following six agronomic traits were measured: fruit length, fruit width, length/width ratio, number of fruits per plant, fruit weight and plant yield. Finally, an earliness index was calculated, defined as the number of days after sowing in which 50% of the total yield (by weight) was harvested. The analysis of variance (ANOVA) and the LSD (Least Significant Difference) *post hoc* test were performed using the agricolae R package (de Mendiburu, 2021). The correlation matrix among four agronomic traits (fruits per plant, fruit weight, plant yield and earliness index) was performed using the Hmisc R package (Harrell, 2025).

## 3 Results

### 3.1 Collection, multiplication and conservation of vegetable melon germplasm

**Figure 1:**
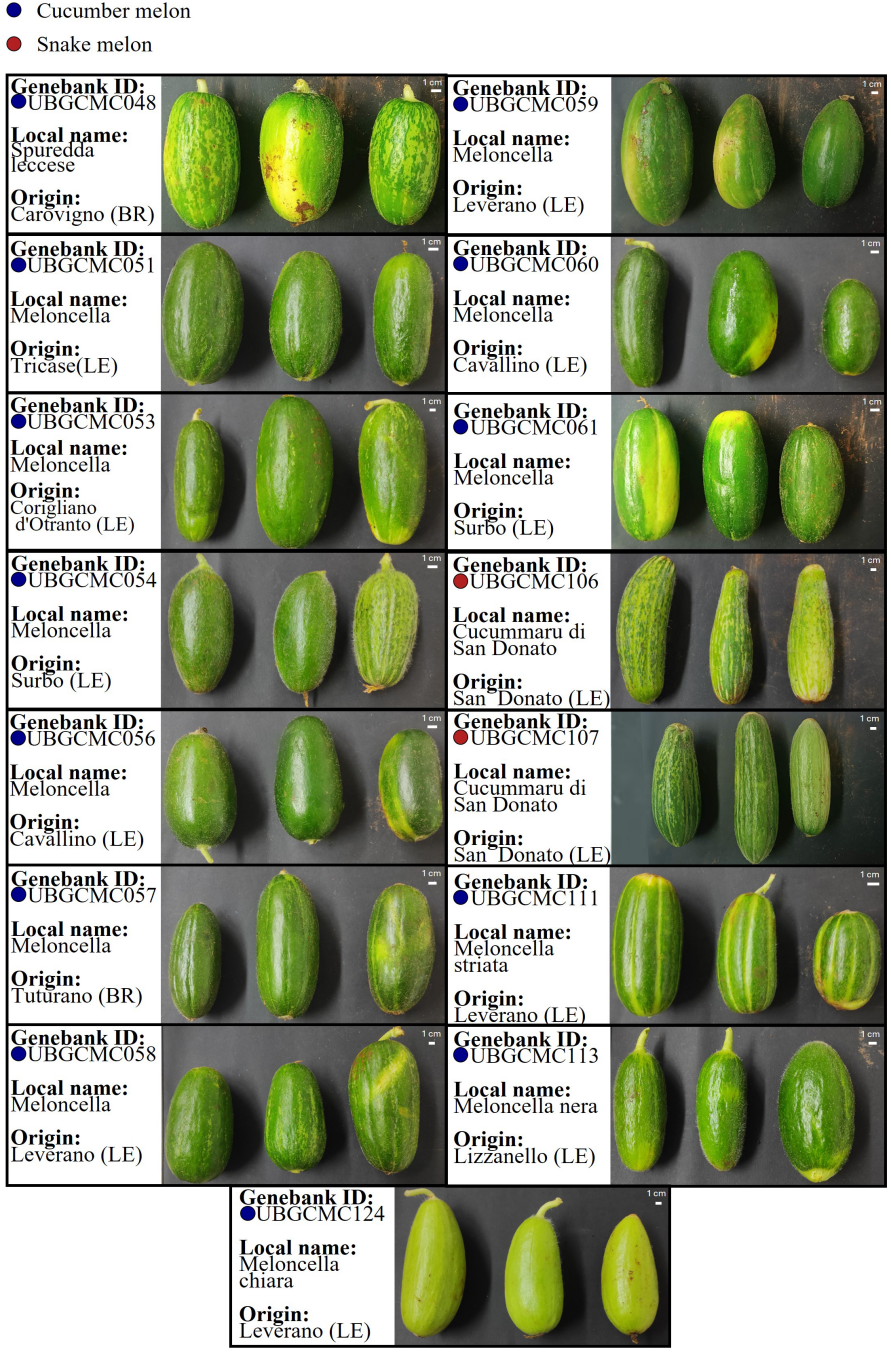
Phenotype of *Cucumis melo* populations collected in this study. For each population, information is provided on the ID code at the UNIBA Genebank, and the municipality of origin in the province of Lecce (LE) or Brindisi (BR), within the Salento area. Photos refer to pepos collected from three randomly selected plants. Cucumber melon and snake melon populations are indicated by blue and red dots, respectively.

In total, thirteen farmer-maintained cucumber melon populations and two snake melon populations were collected and successfully multiplied under controlled conditions (**Figure 1**; **Supplementary Figure 1**). Seed weight obtained by each population ranged from 50g to 450g. All the materials were transferred and catalogued at the UNIBA Genebank.

### 3.2 Whole genome resequencing

**Figure 2:**
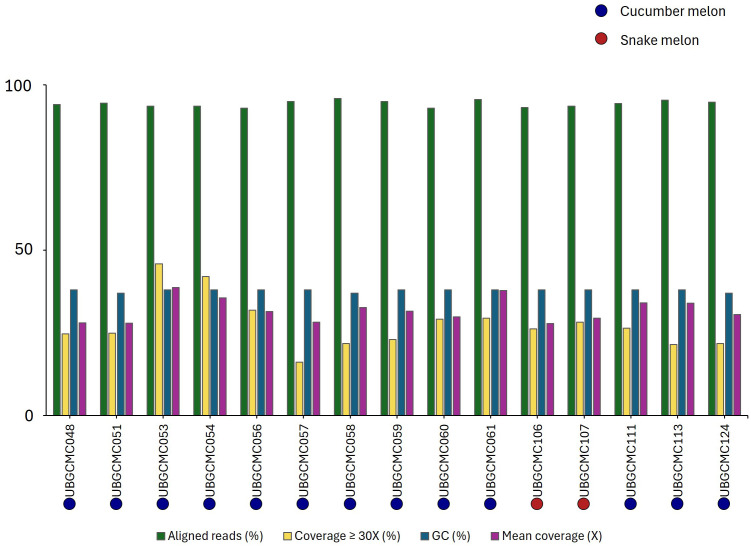
Pool-seq statistics. For each population, the percentage of aligned reads, the genome fraction having a coverage of at least 30x, the GC content percentage and the overall mean coverage are indicated by bars of different colors. Cucumber melon and snake melon populations are indicated by blue and red dots, respectively.

Paired-read genomic resequencing generated a mean value of 13.9 Gb of sequence data per population, ranging from 12.18 Gb to 16.96 Gb. The mean read depth was 31.8x, spanning from 27.8x to 38.7x (**Figure 2**). After the quality control procedure, a variant call format (vcf) file, containing 463,290 SNPs, was obtained.

### 3.3 Intra- and interpopulation diversity

**Figure 3:**
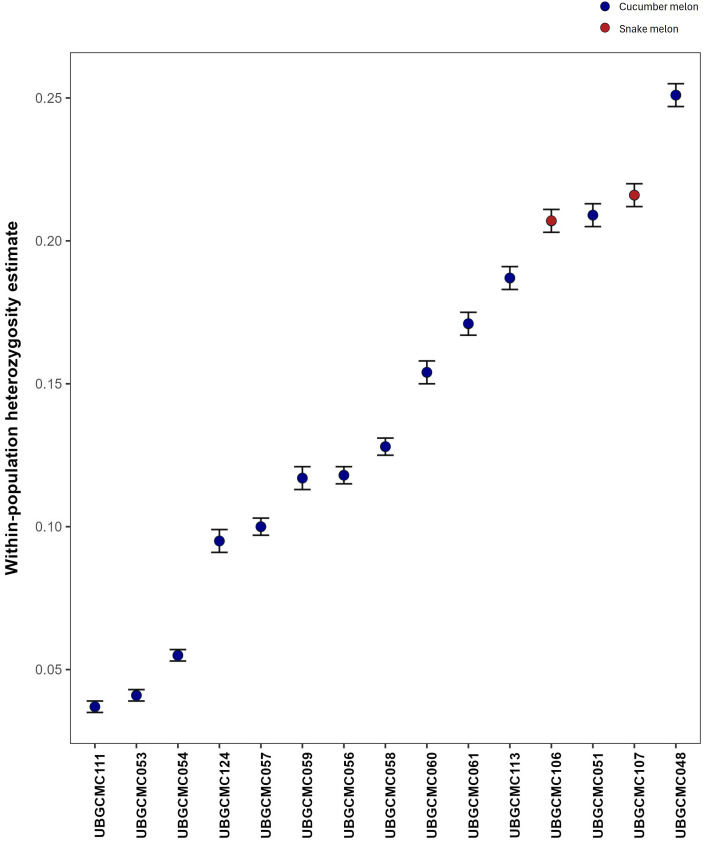
Error-bar plot of heterozygosity estimates. For each population, heterozygosity estimate is represented by a dot, whereas ± SD are indicated by bars. Dots are coloured in blue and red for cucumber melon and snake melon populations, respectively.

The populations UBGCMC111, UBGCMC053, UBGCMC054 and UBGCMC124 showed the highest level of genetic uniformity, with respective heterozygosity estimates of 3.7%, 4.1%, 5.5% and 9.6%. In contrast, the populations UBGCMC048, UBGCMC107, UBGCMC051 and UBGCMC106 showed the highest genetic diversity, with heterozygosity values ranging from 21% to 25% (**Figure 3**).

**Figure 4:**
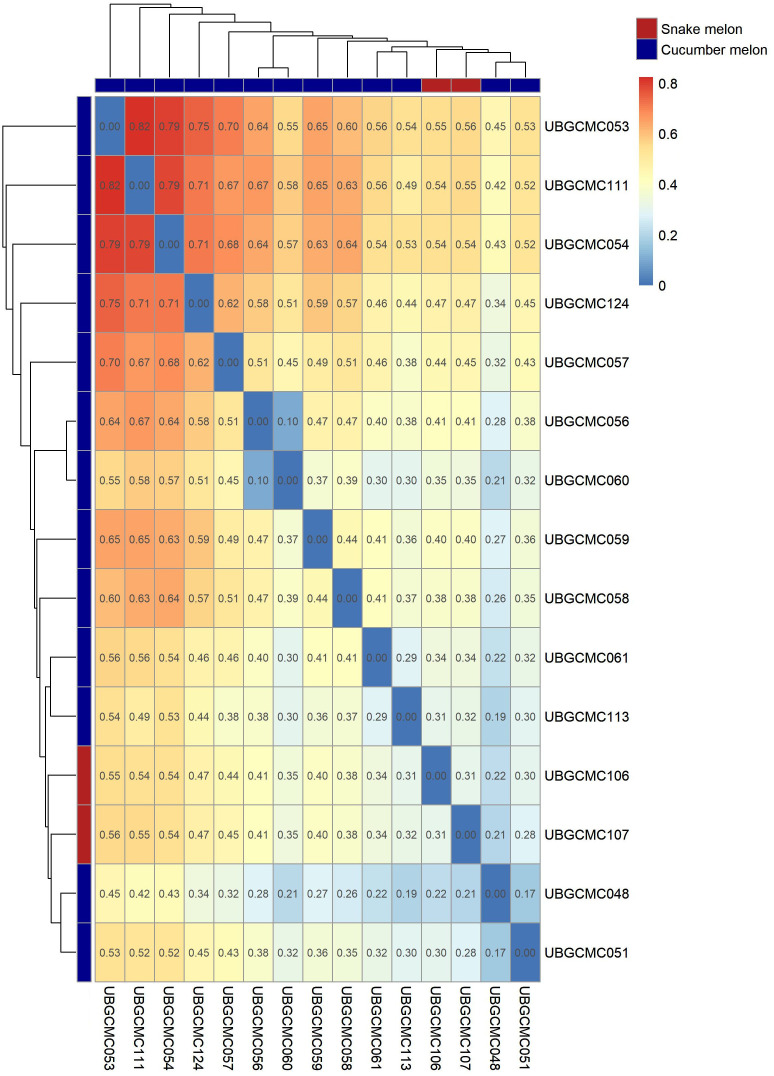
Genetic distance among populations under study. Values in the heatmap refer to pairwise Wright’s F_ST_ index values. The dendrograms at the borders of the heatmap are indicative of genetic distances among populations. Cucumber melon and snake melon populations are indicated by blue and red rectangles, respectively.

A pairwise fixation index (F_ST_) matrix among populations was obtained based on allele frequence estimates (**Figure 4**). Pairwise F_ST_ indices among populations ranged between 0.1 (between the two populations UBGCMC056 and UBGCMC060, both originating from the municipality of Cavallino) and 0.82 (between UBGCMC111 and UBGCMC053).

An F_ST_-based similarity dendrogram was obtained to provide information on genetic relationships among populations (**Figure 4**). The most divergent population was UBGCMC053, followed by UBGCMC111. Notably, the two snake melon populations UBGCMC106 and UBGCMC107 did not form a distinct lineage, suggesting an overall genetic similarity between the *chate* and *flexuosus* botanical varieties.

**Figure 5:**
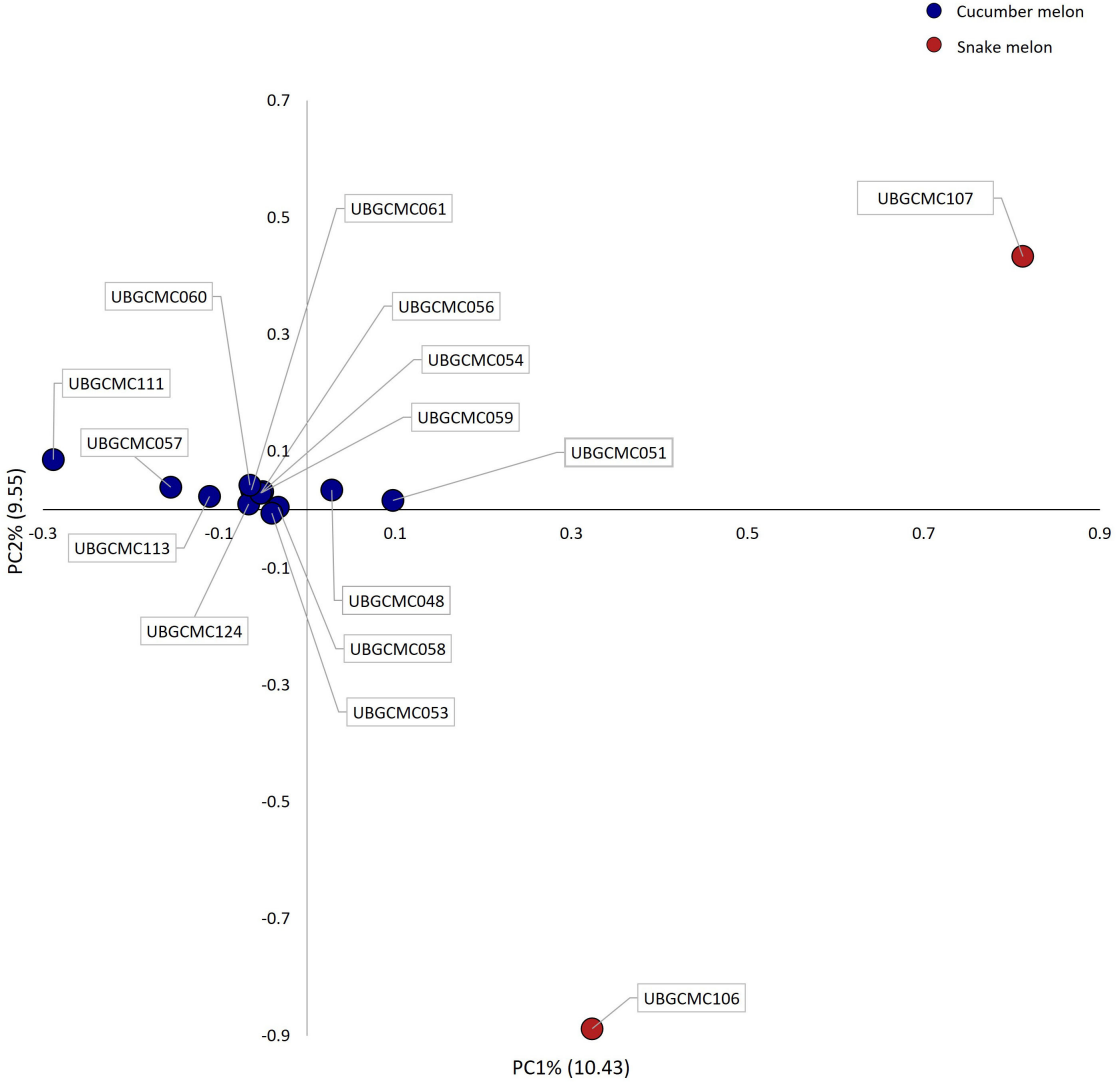
Biplot showing genetic variation explained by the first two principal components (PC1 and PC2). Each dot represents coordinates of individual populations on the two axes. Dots are coloured in blue and red for cucumber melon and snake melon populations, respectively.

To further investigate the genetic structure, PCA was performed (**Figure 5**). The first and the second principal components (PC1 and PC2) clearly differentiated snake melon from cucumber melon populations, which mostly grouped together in the PCA biplot.

### 3.4 Private allele identification and annotation

A total of 1,307 alleles were found to be fixed and private to different populations (i.e. fixed at frequency 1 in a given population and 0 in all others) (**Supplementary Table 1**). Using the Variant Effect Predictor (VEP) tool, we associated fixed and private variants of UBGCMC111 and UBGCMC124, two populations clearly distinguishable from all others by their peculiar pepo phenotype (**Figure 1**; **Table 1**), with 76 and 62 genes, respectively (**Supplementary Tables 3**, **4**).

**Table 1:** Comparison of vegetable melon populations for seven morphological descriptors: fruit color intensity, spot density, spot size, peduncular site shape, distal end shape, grooves and grooves depth.

Gene bank ID	Fruit color intensity	Spot density	Spot size	Peduncular site shape	Distal end shape	Grooves	Grooves depth
UBGCMC048	Medium	High/None	Medium/None	Flat	Round	Few/None	Very shallow/None
UBGCMC051	Dark	None	None	Round	Flat	None	None
UBGCMC053	Dark	Low	Small	Round	Round	Few	Very shallow
UBGCMC054	Dark	None/High	None/Medium	Peaked	Round	None/Few	None/Shallow
UBGCMC056	Dark	None	None	Flat	Flat/Round	None	None
UBGCMC057	Dark	None	None	Round	Round	Few	Shallow/Medium
UBGCMC058	Dark	Low	Small	Round	Round	Few	Shallow
UBGCMC059	Dark	None	None	Round	Round	Few	Very shallow
UBGCMC060	Dark	None	None	Round	Round	None/Few	None/Very shallow
UBGCMC061	Medium/Dark	None	None	Round	Round	Few/Abundant	Shallow/Very shallow
UBGCMC106	Medium	High	Small/Medium	Round	Round	Few/Abundant	Shallow/Medium
UBGCMC107	Medium/Dark	Medium/High	Small/Medium	Peaked/Round	Peaked/Round	Few/Abundant	Shallow/Medium
UBGCMC111	Dark	None	None	Round	Round	Abundant	Deep
UBGCMC113	Dark	None	None	Peaked/Round	Peaked/Round	Few/None	None/Very shallow
UBGCMC124	Light	None	None	Round/Peaked	Round/Peaked	Few	Shallow

The presence of non-unique phenotypes reflects the phenotypic variation observed within some populations.

### 3.5 Phenotypic characterization

Populations varied in their level of uniformity for fruit morphological descriptors (**Figure 1**; **Table 1**). Fruit color intensity varied from pale to dark green, with the pale green phenotype observed exclusively in UBGCMC124. Most populations lacked rind spots, while a few showed variable spot size and density. The peduncular site and distal end were categorized as flat, round and peaked. Longitudinal grooves were absent or shallow in most populations, with deep grooves only observed in UBGCMC111.

Regarding quantitative traits, ANOVA revealed a significant effect of the population in all cases. Growing season variation (with data on temperature and rainfall reported in **Supplementary Figures 2**, **3**) also significantly affected all the traits, except for the number of fruits per plant. Population-by-year interaction was always non-significant, except for fruit length (**Supplementary Table 2**).

As expected, the fruit length/width ratio was higher in the two *C. melo* var. *flexuosus* populations (4.56 for UBGCMC106 and 2.94 for UBGCMC107) than in the *C. melo* var. *chate* populations, which ranged from 2.05 (UBGCMC058) to 2.75 (UBGCMC113) (**Table 2**). A wide variation was observed in the number of fruits per plant, ranging from 5.72 (UBGCMC054) to 14.58 (UBGCMC060), whereas fruit weight showed moderate variation, ranging from 0.21 kg (UBGCMC056) to 0.32 kg (UBGCMC051, UBGCMC060, and UBGCMC113) (**Table 2**). Finally, the earliness index ranged from 37.67 days (UBGCMC056) to 51.67 days (UBGCMC113) (**Table 2**). Correlation analysis among four agronomic traits (**Table 3**) indicated positive and significant correlation between fruits per plant and plant yield.

**Table 2:** Comparison of vegetable melon populations for fruit length, fruit width, length/width ratio, fruits per plant, fruit weight, plant yield and earliness index.

Genebank ID	Fruit length (cm) 2023	Fruit length (cm) 2024	Fruit width (cm)	Length/width ratio	Fruits per plant	Fruit weight (Kg)	Plant yield (Kg)	Earliness index*
UBGCMC048	10.98 ± 2.23^cde^	11.88 ± 3.05^efg^	4.95 ± 1.17^bc^	2.44 ± 0.66^def^	8.75 ± 3.5^def^	0.27 ± 0.03^abc^	2.39 ± 1.06^de^	49.33 ± 3.08^ab^
UBGCMC051	10.32 ± 1.14^def^	11.76 ± 2.39^efg^	5.38 ± 1.32^abc^	2.17 ± 0.38^gh^	6.83 ± 2.71^ef^	0.32 ± 0.05^a^	2.13 ± 0.83^de^	45.5 ± 4.59^abcd^
UBGCMC053	12.99 ± 1.5^b^	13.28 ± 2.33^bcd^	6.25 ± 6.31^a^	2.52 ± 0.54^cd^	11.2 ± 1.73^abcd^	0.28 ± 0.04^abc^	3.15 ± 0.73^abcd^	45.0 ± 5.59^abcd^
UBGCMC054	9.09 ± 3.23^f^	13.36 ± 2.27^bcd^	6.12 ± 2.13^a^	2.15 ± 0.61^gh^	5.7 ± 2.9^f^	0.31 ± 0.05^ab^	1.87 ± 1.27^e^	41.17 ± 3.82^de^
UBGCMC056	9.56 ± 0.65^ef^	10.37 ± 1.27^h^	4.9 ± 0.77^bcd^	2.11 ± 0.39^gh^	10.37 ± 5.11^bcde^	0.21 ± 0.11^d^	2.67 ± 1.3^cde^	37.67 ± 18.8^e^
UBGCMC057	12.33 ± 1.29^bc^	11.29 ± 2.09^fgh^	4.85 ± 1.08^bcd^	2.42 ± 0.3^def^	9.13 ± 2.83^cdef^	0.28 ± 0.03^abc^	2.52 ± 0.86^de^	42.17 ± 3.97^cde^
UBGCMC058	11.09 ± 1.2^cde^	10.22 ± 1.83^h^	5.18 ± 1.07^bc^	2.05 ± 0.32^h^	14.03 ± 3.85^ab^	0.27 ± 0.01^abc^	3.8 ± 1.0^abc^	45.0 ± 5.06^abcd^
UBGCMC059	11.49 ± 1.8^bcd^	12.17 ± 2.06^def^	5.05 ± 1.09^bc^	2.45 ± 0.51^def^	10.67 ± 4.12^abcde^	0.30 ± 0.04^abc^	3.22 ± 1.47^abcd^	47.67 ± 2.34^abcd^
UBGCMC060	11.09 ± 1.2^cde^	11.85 ± 2.02^efg^	5.08 ± 0.97^bc^	2.36 ± 0.49^defg^	14.58 ± 4.01^a^	0.28 ± 0.04^abc^	4.09 ± 1.51^a^	47.5 ± 8.98^abcd^
UBGCMC061	12.38 ± 0.96^bc^	12.71 ± 2.8^cde^	5.65 ± 1.06^ab^	2.25 ± 0.24^fgh^	12.82 ± 1.64^abc^	0.32 ± 0.05^a^	4.06 ± 0.9^ab^	44.83 ± 4.26^bcd^
UBGCMC106	15.67 ± 3.19^a^	17.77 ± 3.28^a^	4.01 ± 1^d^	4.56 ± 1.38^a^	9.77 ± 2.01^cde^	0.27 ± 0.03^abc^	2.65 ± 0.49^cde^	43.0 ± 5.59^bcde^
UBGCMC107	17.17 ± 1.66^a^	13.78 ± 2.63^bc^	5.07 ± 1.15^bc^	2.94 ± 0.56^b^	9.41 ± 2.87^cdef^	0.30 ± 0.05^abc^	2.82 ± 1.05^bcde^	47.17 ± 1.6^abcd^
UBGCMC111	10.56 ± 1.1^def^	10.95 ± 1.75^gh^	4.9 ± 1.01^bcd^	2.28 ± 0.44^efgh^	14.53 ± 2.69^a^	0.26 ± 0.03^bc^	3.87 ± 1.02^abc^	47.83 ± 2.48^abcd^
UBGCMC113	10.68 ± 1.02_cdef_	13.97 ± 3.2^b^	5.04 ± 1.27^bc^	2.75 ± 0.65^bc^	8.53 ± 3.35^def^	0.32 ± 0.07^a^	2.78 ± 1.44^cde^	51.67 ± 5.54^a^
UBGCMC124	11.34 ± 1.06^cd^	11.25 ± 1.75^fgh^	4.64 ± 1.04^cd^	2.51 ± 0.47^cde^	9.0 ± 4.65^cdef^	0.25 ± 0.04^cd^	2.33 ± 1.45^de^	48.5 ± 9.48^abc^
Mean	11.78	12.4	5.14	2.53	10.35	0.28	2.96	45.6

Data are presented as mean ± standard deviation of biological replicates for individual years in the case of fruit length, for which significant genotype:year interaction was detected, or as mean ± standard deviation of biological replicates across the two years for all the other traits, for which no significant genotype:year interaction was observed.

*Days from sowing date

Means not associated with the same letter are significantly different at *p* < 0.05 (LSD test).

**Table 3:** Correlation matrix among four agronomic traits (fruits per plant, fruit weight, plant yield and earliness index) recorded in this study.

Trait Trait	Fruits per plant	Fruit weight	Plant yield	Earliness index
Fruits per plant	1	-0.33	0.91*	-0.04
Fruit weight		1	0	0.04
Plant yield			1	0.14
Earliness index				1

The asterisk indicates statistically significant correlations (*p* < 0.05).

## 4 Discussion

Cucumber melon, once widespread in the Mediterranean Basin, has seen its cultivation to decline drastically, and is now mainly grown in the Salento area of Southern Italy. The germplasm collection presented here is therefore of pivotal importance for preserving the cucumber melon gene pool from further genetic erosion. In addition, it provides a valuable foundation for breeding programs in cucumber melon, which is regaining popularity in both national and international markets, as well as in other *C. melo* taxonomical groups, including sweet melons.

The pool-seq approach applied in this study has confirmed to be a valuable and cost-effective approach for generating population-level genetic data, despite intrinsic limitations such as lack of individual genotype resolution and potential allele frequency biases. Estimation of heterozygosity indicated a highly variable level of genetic diversity within the populations under study (**Figure 3**). The populations UBGCMC111 and UBGCMC124, characterized by clearly distinguishable pepos (deeply grooved and pale green, respectively, **Figure 1**), were among those with the lowest heterozygosity, likely reflecting stronger phenotypic selection exerted by farmers.

Analysis of genetic diversity by principal component analysis (PCA) revealed a relatively low amount (about 20%) of total genetic variation explained by the first two components, suggesting that genetic differentiation among the studied populations is driven by many loci with small differences in allele frequency. Nonetheless, the PCA biplot highlighted specific patterns of variation for the two snake melon populations UBGCMC106 and UBGCMC107, consistent with their distinct morphological features.

Notably, hierarchical clustering using Wright’s fixation index (F_ST_) revealed no genetic divergence between snake melon and cucumber melon populations, as they clustered together (**Figure 4**). This result aligns with previous reports of genetic and physico-chemical similarity between the two botanical varieties *chate* and *flexuosus* (Ali-Shtayeh et al., 2017; Flores-León et al., 2022; Branca et al., 2023). Given their shared cultivation history, dating back to Ancient Egypt, and their mention as *qishu’im* in Biblical texts (Janick et al., 2007), our evidence supports the hypothesis that snake melon originated from cucumber melon or vice versa. However, as we only analyzed two snake melon populations, we cannot exclude the possibility that these represent hybrids between the *chate* and *flexuosus* groups. The pepos of UBGCMC106 and UBGCMC107 share striking similarity with snake melons traditionally cultivated in Israel and the Palestinian territories, known as *Green Baladi* or *Baladi Akhder* (Ali-Shtayeh et al., 2017; Omari et al., 2018).

According to F_ST_-based hierarchical clustering, the most divergent population is UBGCMC053, originating from the Municipality of Corigliano d’Otranto (**Figure 2**). Remarkably, Corigliano d’Otranto locates in a culturally and linguistically unique area in Salento known as Grecìa Salentina, which has deep historical ties to Greek culture, dating back to ancient Magna Graecia and the Byzantine period (Pantaleo, 2006; Guardiano and Stavrou, 2021). It might therefore be speculated that this population derives from a distinct introduction from the Greek Peninsula. UBGCMC111, the second-most divergent population in the panel, might also originate from a different gene pool, as it is characterized by unique pepo morphological features.

The identification of fixed and private DNA variants in most of the populations under study represents a valuable resource for their traceability and valorization. In the population UBGCMC111, currently the most widely cultivated and marketed, one fixed private variant was associated with a 3’-UTR mutation in a gene of the ethylene-insensitive 3 (EIN3) family, involved in ethylene signaling. Another variant was annotated in the downstream region of a gene belonging to the *Lateral Organ Boundaries* (*LOB*) family, which plays a major role in melon fruit development and morphogenesis (Tufekci, 2023). In UBGCMC124, fixed private intronic and downstream gene mutations were associated with genes putatively involved in photosynthesis, encoding a chloroplast pheophytinase and a photosystem II D1 precursor processing protein (PSB27-H2), respectively. Interestingly, pheophytinases are involved in chlorophyll breakdown (Schelbert et al., 2009), which could explain the distinctive pale green pepo phenotype observed in this population (**Figure 1**). Further functional studies, based on targeted mutagenesis or transgenic expression, may prove causal association between mutations in the candidate genes above mentioned and the observed phenotypic variation.

With respect to bio-agronomic traits, significant differences were observed among the populations, in line with the results of the genetic analyses. In addition, year appeared as a significant source of variation, likely due to peculiar climatic conditions (**Supplementary Figures 2**, **3**). In contrast, the population:year interaction resulted to be non-significant in most cases,5 suggesting that all populations may exhibit a relatively stable response to different environmental conditions. Plant yield was significantly associated with the number of fruits, but not with fruit weight (**Table 3**). As expected, the population UBGCMC111, currently the most popular among farmers, also exhibited good agronomic performance in terms of yield and earliness. However, several other populations were also ranked among the best performing (**Table 2**). Overall, the cucumber melon and snake melon populations described in this study are the result of farmer selection under local conditions and are, in most cases, genetically heterogeneous. We therefore suggest that formal breeding programs, leveraging the genetic and phenotypic information presented here, could achieve significant genetic gains in the short term.

## Data Availability

The datasets presented in this study can be found in online repositories. The names of the repository/repositories and accession number(s) can be found below: https://www.ncbi.nlm.nih.gov/, PRJNA1260727.
