# Supplementary material for: Whole-genome sequencing and phenotyping of neglected and underutilized vegetable melons from the Salento diversity centre (Southern Italy)
Source: Front Plant Sci. 2025 Aug 19;16:1644621. doi: 10.3389/fpls.2025.1644621 (PMC12401921; doi:10.3389/fpls.2025.1644621)
Supplement: Supplementary file 1 [file DataSheet1.docx]

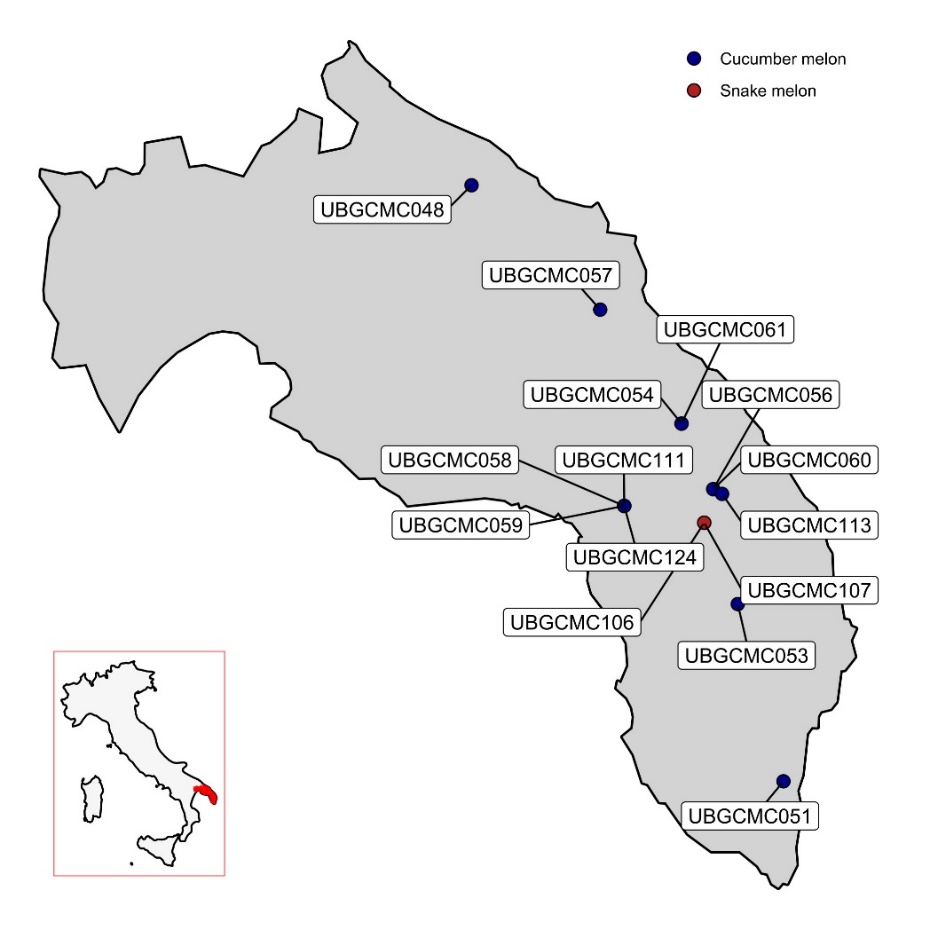


**Supplementary Figure 1.** Geographical origin of the 15 vegetable melon populations collected in this study from the Salento area (Southern Italy). Cucumber melon and snake melon populations are indicated by blue and red dots, respectively.

**Supplementary Figure 2.** Climatic data for the field trial carried out in 2023.

**Supplementary Figure 3.** Climatic data for the field trial carried out in 2024.
